# Renal transplantation and variations in blood supply: a narrative review

**DOI:** 10.3389/frtra.2026.1843066

**Published:** 2026-07-06

**Authors:** Spencer Thrailkill, Abduelmenem Alashkham, Mohamed Elajnaf, Tarek Almabrouk

**Affiliations:** 1 Edinburgh Medical School, Department of Anatomy, University of Edinburgh, Edinburgh, United Kingdom; 2 Zawia Faculty of Medicine, University of Zawia, Zawia, Libya; 3 Urology Department, Ipswich Hospital, Ipswich, United Kingdom; 4 St. George’s International School of Medicine, Northumbria University, Newcastle, United Kingdom

**Keywords:** renal anatomy, renal artery variation, renal tranplantation, renal vascular variation, transplantation

## Abstract

**Introduction:**

Chronic kidney disease (CKD) has become a significant global health challenge as patients frequently progress to end-stage renal disease leading to kidney transplantation. Anatomical variations of the renal vascular structure may affect donor candidates, surgical management, and graft success, making it important to consider in transplantation. Although the use of anatomically complicated grafts is growing, differences in vascular pattern continue to impact clinical decision-making.

**Objective:**

To investigate the anatomical features of the renal vasculature and determine its clinical significance for kidney transplantation and their effect on surgical complexity and transplant outcomes.

**Methods:**

Narrative literature review was performed through PubMed, Scopus and Google Scholar. Articles were included if they were related to kidney vascular anatomy, imaging evaluation, surgical methods, and transplant results. Literature: Included in this review were historical and relevant secondary studies to offer a global perspective of the latest developments in renal transplantation practice.

**Results:**

A large body of studies have explored these renal vascular variations with up to 30% of people having at least two renal arteries, and venous variations such as retroaortic or circumaortic renal veins. These variations can lead to a more complex surgery and duration of operation, as well as a higher chance of development of early complications (delayed graft function or vascular thrombosis) due to ischemia-induced complications. Yet evidence is currently accumulating that, with sound preoperative imaging and surgical precision, the long-term prognosis of grafts and patient outcomes is similar to that of standard vascular anatomy graft. Developments in imaging modalities, especially computed tomography angiography, have greatly advanced both preoperative detection and surgical planning.

**Conclusion:**

Such renal vascular variations should not automatically preclude transplantation and should not be considered as an adverse event when donating or transplanting kidneys. Anatomically complex grafts can be successfully used, with careful preoperative assessment, multidisciplinary planning and modern surgical techniques. More standardized reporting and more recent studies are required to optimise donor utilisation and thereby optimize transplant success.

## 1 Introduction

### 1.1 Chronic kidney disease

Chronic kidney disease (CKD) is a progressive condition characterised by impaired renal function, which may ultimately lead to end-stage renal disease (ESRD), requiring renal replacement therapy in the form of dialysis or kidney transplantation (1–5). It represents a major global health burden, affecting over 800 million individuals worldwide, with an estimated prevalence of 11%–13% (6–8). The increasing incidence of CKD is largely driven by diabetes, hypertension, obesity, and an ageing population (8). The number of patients with CKD who require renal replacement therapy grows as CKD continues to progress, placing significant pressure on healthcare systems worldwide (9–11). This growing burden highlights the need for effective treatment strategies and improved access to definitive therapies.

Organ transplantation is an effective treatment for patients with ESRD; however, limited organ availability necessitates careful selection of both recipients and donors to ensure optimal outcomes (12). Recipient eligibility is typically based on advanced renal failure requiring dialysis or significantly reduced renal function, while exclusion criteria vary between transplant programmes and may include severe comorbidities or conditions limiting surgical suitability (12–14). Similarly, donor selection is subject to strict criteria, with kidneys obtained from either deceased or living donors who must demonstrate adequate renal function and overall health (15–17). Despite increasing evidence supporting the safety of transplantation in the presence of renal vascular variations, donor acceptance remains inconsistent across transplant centres, reflecting differences in surgical expertise and risk tolerance (18). Many centers remain reluctant to accept kidneys with multiple arteries, highlighting how anatomical variation continues to influence donor utilisation and clinical decision-making (19).

Kidney transplantation requires coordinated surgical procedures involving both donor and recipient. Advances in minimally invasive techniques, including laparoscopic and robot-assisted donor nephrectomy, have significantly reduced donor morbidity while maintaining procedural effectiveness (20). Preoperative imaging, particularly computed tomography (CT) and magnetic resonance imaging (MRI), plays a critical role in evaluating renal anatomy and identifying vascular patterns prior to surgery, thereby guiding donor selection and operative planning (21).

A key determinant of transplant success is the management of ischemia. Prolonged warm ischemia is associated with delayed graft function and poorer outcomes (22). In anatomically complex grafts, such as those with multiple renal arteries or atypical branching patterns, surgical complexity may increase operative time and technical difficulty. However, when these variations are accurately identified and appropriately managed, long-term outcomes are generally comparable to those of single-vessel grafts (23). These findings emphasise the importance of detailed anatomical knowledge, particularly of renal vascular variation, in optimising surgical outcomes.

Despite these initiatives, the imbalance between organ supply and demand remains a persistent challenge. This has prompted increasing interest in expanding donor utilisation, including the acceptance of kidneys with anatomical variations. Although such variations are relatively common, they are still approached with caution in some centers due to concerns regarding technical complexity and potential effects on graft function.

In this context, an understanding of renal structure and function is essential. The kidneys are responsible for maintaining metabolic and physiological homeostasis through the excretion of waste products, regulation of fluid and electrolyte balance, and endocrine activity (24). These functions are carried out at the level of the nephron, where glomerular filtration and tubular processing allow for the selective removal of metabolic by-products while preserving essential solutes (25). Renal function is closely dependent on adequate perfusion and the integrity of the vascular supply. The kidneys receive a substantial proportion of cardiac output, reflecting their high metabolic activity. Importantly, the intrarenal arterial system is organised into segmental branches that function as end arteries, with limited collateral circulation. As a result, any variation in vascular anatomy may have direct implications for both renal function and surgical management.

These considerations are particularly relevant in renal transplantation, where precise identification and preservation of vascular structures are critical. Anatomical variations may influence donor selection, operative planning, and the technical aspects of vascular anastomosis. However, with advances in preoperative imaging and surgical technique, many of these challenges can be effectively managed, supporting the use of anatomically complex grafts in appropriate settings.

The current literature contains numerous studies describing renal vascular anatomy; however, a comprehensive synthesis of these variations and their clinical implications in transplantation remains limited. Therefore, this review aims to examine the anatomical and clinical evidence surrounding renal vascular variations, their impact on renal transplantation, and the strategies used for their identification and surgical management.

## 2 Method

This study was conducted as a narrative review to examine the anatomical and clinical significance of renal vascular variations in kidney transplantation. The structure and reporting of the review were informed by the SANRA (Scale for the Assessment of Narrative Review Articles) recommendations to improve methodological transparency. As this was a narrative review rather than a systematic review, the search strategy was designed to provide a broad and clinically relevant overview of the literature rather than an exhaustive systematic synthesis.

A literature search was performed using PubMed, Scopus, and Google Scholar between January 2025 and March 2025. The search included studies published between 1980 and 2025 to incorporate both historical and contemporary perspectives on renal transplantation, vascular anatomy, imaging modalities, and surgical techniques.

Search terms included combinations of: “renal vascular variation,” “multiple renal arteries,” “accessory renal artery,” “renal vein anomalies,” “retroaortic renal vein,” “circumaortic renal vein,” “kidney transplantation,” “living donor nephrectomy,” “vascular reconstruction,” and “transplant outcomes.” Boolean operators (AND/OR) were used to refine the search strategy. Where applicable, Medical Subject Headings (MeSH) terms including “Kidney Transplantation,” “Renal Artery,” “Renal Veins,” and “Anatomic Variation” were explored in PubMed.

Articles were considered eligible if they were published in English and addressed renal vascular anatomy, embryology, imaging assessment, surgical management, or transplant-related outcomes in human subjects. Priority was given to clinically relevant anatomical studies, imaging-based investigations, systematic reviews, meta-analyses, and landmark studies relevant to current transplant practice. Studies focusing exclusively on non-clinical experimental models, unrelated renal pathology, or articles lacking relevance to kidney transplantation were excluded.

Studies were selected based on their relevance to the objectives of the review, particularly regarding:

- Anatomical classification of renal vascular variations
- Embryological basis of vascular variation
- Preoperative imaging and diagnostic assessment
- Surgical planning and vascular reconstruction
- Short- and long-term transplant outcomes

The findings were narratively synthesised to provide an integrated overview of the current evidence and its clinical relevance to contemporary kidney transplantation practice.

## 3 Normal anatomy and physiology of the renal vasculature

The kidneys maintain metabolic homeostasis through excretion of waste products, regulation of fluid and electrolyte balance, acid–base control, endocrine activity, and contribution to hemodynamic stability (21). These functions depend on an adequate and well-organised vascular supply. At the microscopic level, the nephron is the functional unit of the kidney and comprises a glomerulus and a tubular system. Glomerular filtration is driven by hydrostatic pressure, permitting passage of water and small solutes while retaining larger molecules such as proteins and blood cells (25). The filtrate is subsequently modified by tubular reabsorption and secretion, allowing conservation of essential water, electrolytes, and nutrients, while facilitating excretion of metabolic waste products including urea and creatinine (25). The kidneys also regulate sodium and water balance, thereby influencing blood volume and blood pressure, and secrete hormones such as renin and erythropoietin, in addition to activating vitamin D (25, 26).

In classical anatomy, each kidney is supplied by a single renal artery, which is a lateral branch of the abdominal aorta. These vessels usually arise at the intervertebral disc level between the lumbar vertebrae L1 and L2, just below the origin of the superior mesenteric artery (27). The kidneys receive 20%–25% of the cardiac output via the two renal arteries (28). The left renal artery typically starts slightly higher than the right renal artery does. The right renal artery was longer and passed posterior to the inferior vena cava. Each renal artery divides into anterior and posterior branches as it approaches the renal hilum. These divisions supply the renal parenchyma and further divide it into segmental arteries (29). There were five renal arterial segments: apical, upper, middle, lower, and posterior. These supply each segment of the kidney as “end arteries” and do not anastomose with the adjacent branches. This means that the area supplied by an individual segmental branch can be resected surgically without any risk of infarction in the parenchyma supplied by another segmental artery (30). The lobar arteries are the first branches of the segmental arteries and typically supply one branch to each renal pyramid. Prior to reaching the renal pyramid, the lobar arteries are further divided into two or three interlobar arteries that surround the cortex of each pyramid to supply the kidney (31, 32). The interlobar arteries then branch into the arcuate arteries at the renal cortex/medulla junction. As they travel between the renal cortex and medulla, the arcuate arteries branch into the interlobular arteries that ascend radially into the cortex. Next, the interlobular arteries form afferent glomerular arterioles that supply the glomerulus and use their diameter to control the Glomerular Filtration Rate (GFR). The glomerulus filters blood forms the filtrate, and is the first step in the capillary-nephron portal system. When plasma filters from the glomerulus into Bowman's space are termed filtrates, they are excreted from the body (33). The remaining blood leaves the glomerulus via the efferent glomerular arterioles. Efferent glomerular arterioles transport blood away from the glomerulus and, like afferent arterioles, can control GFR according to their diameter (34, 35).

It is important to note that two types of nephrons exist, cortical and juxtamedullary (36). Cortical nephrons have short loops of Henle and penetrate only the outer medulla. Juxtamedullary nephrons have long loops of Henle that penetrate deep into the inner medulla (29). Each capillary-nephron portal system consists of two steps. The first step for both nephron types was filtration via the glomerulus. For cortical nephrons, the second step involves the efferent arterioles delivering the blood to the peritubular capillaries. Peritubular capillaries are located around the renal tubules and perform reabsorption and secretion (37). During reabsorption, water, sodium, and glucose cross the lumen via the renal tubule cells back into the peritubular capillaries. Conversely, substances such as ammonia or potassium are selectively transported from the peritubular capillaries to the lumen via the same renal tubule cells for excretion (36). In juxtamedullary nephrons, the second step is the efferent arteriole delivering blood to the vasa recta. The vasa recta functions similarly to peritubular capillaries, performing reabsorption and secretion similarly. However, juxtamedullary nephrons penetrate the inner medulla and account for only 15% of the total nephrons (35, 38).

**Figure 1:**
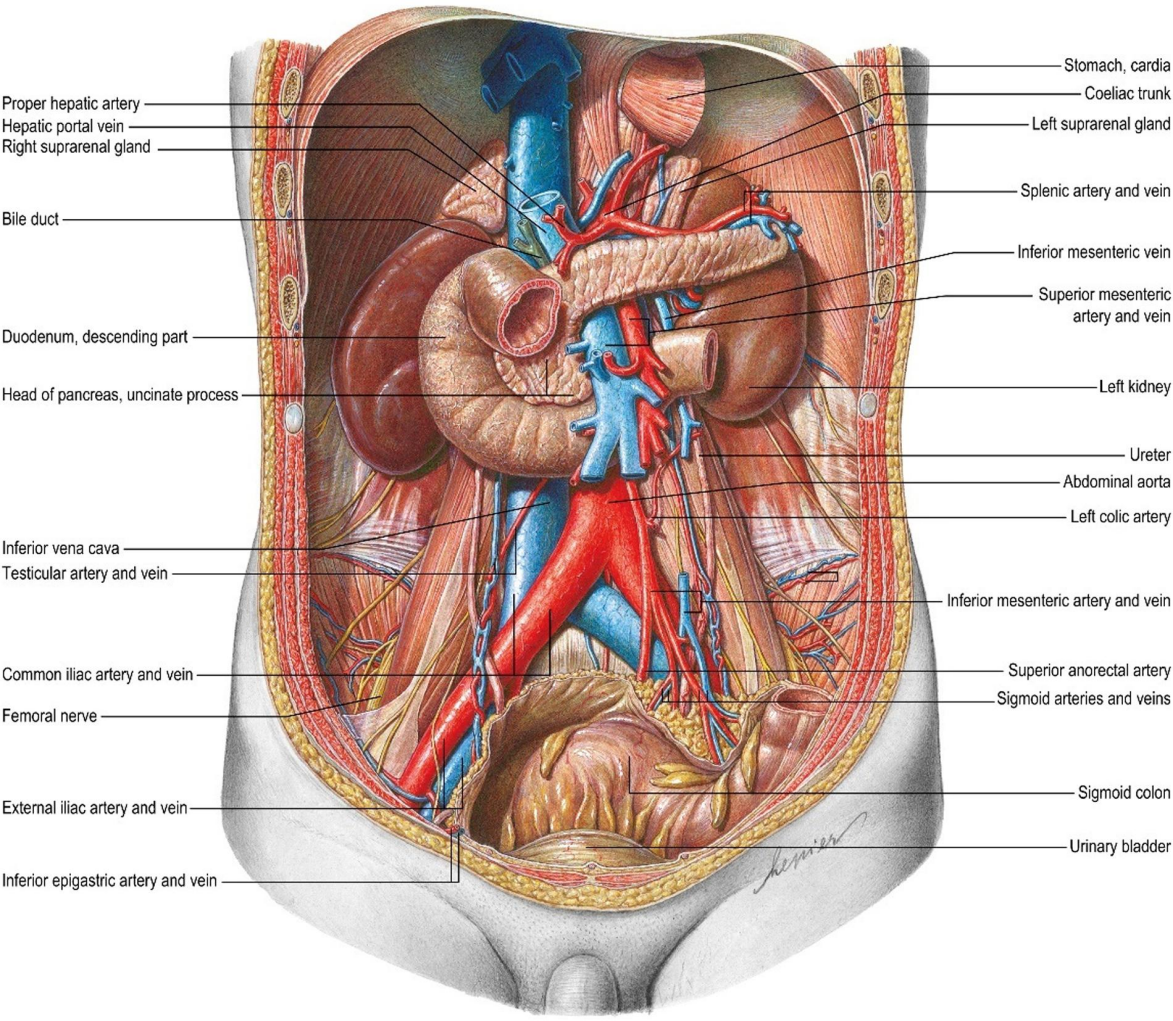
This gross anatomy illustration highlights the relationships of the kidneys with surrounding abdominal organs. Key structures near the kidneys include the right and left suprarenal glands, ureters, and the abdominal aorta. The superior and inferior mesenteric arteries and veins, as well as the renal arteries and veins, demonstrate vascular connections. Other nearby structures include the inferior vena cava, duodenum, pancreas, spleen, and the common iliac arteries and veins, showing the complex network of organs and vessels around the kidneys. Permission to use this figure has been granted (34).

Venous drainage of the kidneys closely mirrors the arterial supply. Both the peritubular capillaries and vasa recta return their blood supply to the venules, which converge to form the interlobular veins. The interlobular veins unite to form arcuate veins, which drain into the interlobar veins before finally forming the renal vein. The renal vein drains into the inferior vena cava, returning blood to the right atrium (39, 40). The renal veins lie anterior to the renal arteries. The left renal vein originates at the hilum of the kidney before running posterior to the splenic vein and body of the pancreas and then across the anterior surface of the abdominal aorta, inferior to the superior mesenteric artery. The left renal vein is typically 8.5 cm, roughly three times longer than the right, with both the left gonadal and left adrenal veins draining into it along its course (40). The right renal vein lies posterior to the descending duodenum and drains directly into the inferior vena cava, typically approximately 2.5 cm (34, 35). The gross anatomy and relationship between the nephrons and renal vasculature are shown in Figure 1.

Ureters arise from the renal pelvis at the hilum of the kidney and descend through the abdomen anterior to the psoas major before crossing the pelvic brim to enter the pelvic cavity. Descending along the pelvic walls, the ureters continue to the bladder wall before piercing obliquely to form a one-way valve (41).

The kidneys were situated on the posterior abdominal wall and positioned behind the peritoneum. These are paired organs located on either side of the vertebral column at the level of the 12th rib. The right kidney was slightly lower than the left kidney because of its proximity to the liver (42). Each kidney has two surfaces, anterior and posterior, separated by lateral and medial borders. The lateral and medial borders converged at the upper and lower poles of the kidney, respectively. The diaphragm covers the upper posterior surface of the kidneys. Additionally, the upper poles of both kidneys adjoined the adrenal glands. The right kidney is anteriorly related to the right colonic flexure, second part of the duodenum, head of the pancreas, and right lobe of the liver, with which it shares the hepatorenal ligament. In contrast, the left kidney is anteriorly related to the spleen, stomach, pancreas, jejunum, descending colon, and left colonic flexure (43). The left kidney is connected to the spleen via a shared lienorenal ligament. The kidneys are encompassed by the perinephric fascia, known as Gerota's fascia, along with adrenal glands and perinephric fat (44). The posterior surface of the kidney lies on the psoas major muscle, causing the hilum of the kidney to rotate anteriorly.

This classical pattern provides the framework for understanding renal vascular variation. Because the kidney is highly perfused and segmentally supplied by end arteries, anatomical variation may affect donor selection, operative planning, vascular reconstruction, and short-term transplant outcomes.

Although this is the traditional anatomy of the kidney, there are numerous variations. Blood supply is highly variable and is a great clinical consideration for transplantation. Undoubtedly, single vascular anatomy is preferred for transplantation. However, recent studies have concluded that given an adequate preoperative evaluation and an experienced surgical team, renal vascular multiplicity should not be viewed as a contraindication for donation (45). This is an important finding as renal vascular variations, particularly multiple renal arteries, are common. In one imaging study of 504 patients, the prevalence of multiple renal arteries was the highest, occurring in 31.3% of patients (46). In the same study, pre-hilar branching of the renal artery occurred in 6.5% of patients. The venous supply also varied, with 21.6% of patients possessing multiple right renal veins. Other venous variations were present with a circumaortic left renal vein in 5.2% of the patients, a retroaortic left renal vein in 4.2%, and 7.3% with late venous confluence.

While operating on these variations is certainly possible, some surgeons may be apprehensive about performing nephrectomy with variations as it comes with inherent risks. Renal vascular variation may increase the likelihood of acute tubular necrosis, reduce graft function, lengthen hospital stay, and even lead to organ rejection (47). Hemorrhagic complications are also notably more common in transplants with numerous renal arteries (48). Furthermore, multiple arteries are a risk factor for renal artery thrombosis, a rare but dangerous postoperative complication (49). Although measures are being taken to reduce post-transplantation complications, postoperative medical and surgical complications are significant sources of morbidity and death associated with the procedure (50).

Medical professionals must identify new methods of using resources in light of the growing organ demand and the increasing number of willing donors. An opportunity to expand the donor pool exponentially by overcoming anatomical limitations exists. To ensure successful transplantation despite these limitations, future operations will require surgeons to be more familiar with the anatomical variations in the renal arterial supply. Surgeons performing kidney transplants must have a thorough understanding of the renal vascular variations.

### 3.1 Renal vascular variations relevant to kidney transplantation

#### 3.1.1 Developmental basis of renal vascular variation

Renal vascular variations arise from embryological development and are highly relevant to kidney transplantation because they influence vascular number, branching pattern, and relationships at the renal hilum. The kidneys arise from the metanephros at approximately week four of gestation. As the embryo grows, the kidneys ascend from the pelvis and undergo medial rotation beginning around week six, allowing the hilum to rotate from an anterior-facing to a medial-facing position. During this ascent, the metanephros are supplied sequentially by arteries arising at progressively higher levels of the aorta, while the more caudal vessels normally regress. By the ninth week of gestation, the kidneys reach the lumbar region, and the most cranial branch becomes the permanent main renal artery (51).

Renal venous variations are similarly rooted in embryological development. The formation of the inferior vena cava and renal veins requires the development, anastomosis, and selective regression of the cardinal, subcardinal, and supracardinal venous systems (52). Persistence or regression of different embryological channels may result in retroaortic, circumaortic, or multiple renal veins. Although the embryological basis of these variants is well established, their clinical importance lies in their effect on donor assessment, operative planning, and vascular reconstruction in transplantation.

#### 3.1.2 Arterial variations

##### 3.1.2.1 Overview of arterial variation

Renal arterial variations are common and directly related to embryological development. The position and source of the blood supply, branching pattern, location of vessel entry into the kidney, and relationship of the renal artery to surrounding structures all depend on developmental events (53). Variations in origin, calibre, and course may occur, including higher or lower aortic origin, differences in length and diameter, and altered relationships with the renal vein or pelvis of the ureter (54). However, from a transplant perspective, branching abnormalities and arterial multiplicity are the most clinically relevant because they may shorten pedicle length, increase the number of anastomoses required, and complicate donor nephrectomy and implantation.

To improve consistency in terminology throughout this review, the principal arterial and venous vascular variants discussed in the literature are summarised in Table 1.

**Table 1:** Anatomical definition of different vascular variations.

Term used	Alternative terms in literature	Working definition
Peri-hilar branching	Early branching, pre-hilar branching	Division of the main renal artery close to the renal hilum or within approximately 15–20 mm from the aortic origin
Multiple renal arteries	Additional renal arteries	Presence of more than one renal artery supplying the kidney
Accessory renal artery	Additional hilar artery, hilar accessory artery	Additional renal artery entering the kidney through the hilum alongside the main renal artery
Aberrant renal artery	Polar artery, upper polar artery, lower polar artery	Additional renal artery entering the kidney directly through the upper or lower pole rather than through the hilum
Retroaortic renal vein	Posterior renal vein	Left renal vein passing posterior to the abdominal aorta
Circumaortic renal vein	Renal venous collar	Presence of anterior and posterior venous channels encircling the aorta

From a transplant perspective, accessory hilar arteries are generally preserved because they contribute directly to hilar perfusion, whereas aberrant or polar arteries require assessment according to calibre, perfusion territory, and reconstructive feasibility. Polar arteries of significant calibre, particularly those greater than 2 mm, are usually preserved where technically feasible to minimise the risk of segmental ischaemia or ureteric complications (55, 56).

##### 3.1.2.2 Peri-hilar and segmental branching patterns

Branching pattern variations include peri-hilar branching, also referred to in the literature as early branching or pre-hilar branching. Terminology remains inconsistent, with little distinction made between these terms across studies. Although definitions vary between studies, early or peri-hilar branching is commonly defined as division of the main renal artery within approximately 15–20 mm of its aortic origin. This variation is clinically important in laparoscopic donor nephrectomy because it may shorten the usable arterial pedicle and create the appearance of multiple arteries during retrieval and reconstruction.

One proposed explanation is persistence of aortic branches that would normally regress during renal ascent. In a study of 1,859 donor nephrectomy candidates, early branching occurred in 6.3% of right renal arteries and 6.5% of left renal arteries (57). In contrast, a study of 248 computed tomography angiographies reported an incidence of 37.3% for single arteries with prehilar branching (58). The wide variation in reported frequency highlights the difficulty of comparing studies that use different definitions. Morphological patterns include duplicated forks, triplicated forks, and ladder patterns (59–61). Although reported incidence varies, peri-hilar branching is common and remains important because it may reduce usable arterial length and affect anastomotic options.

**Figure 2:**
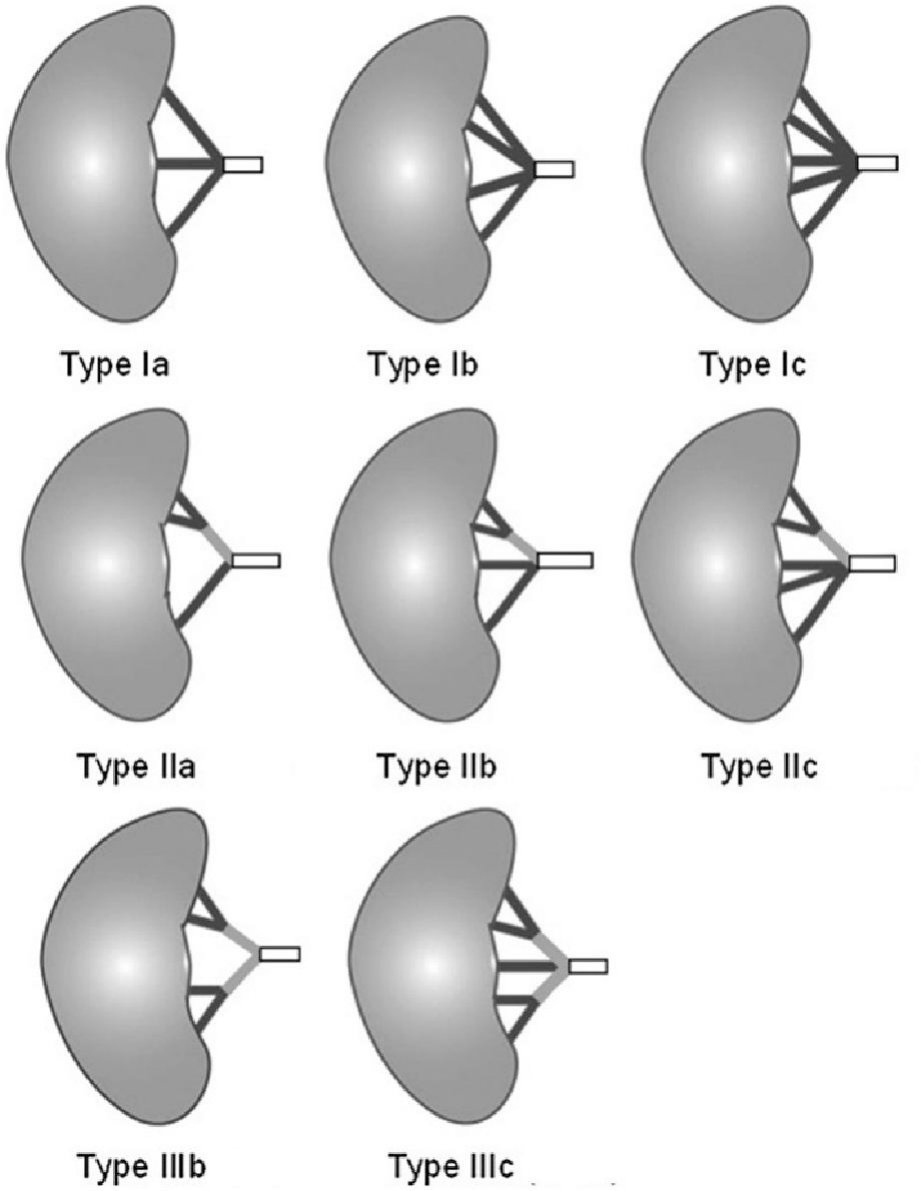
Illustration and classification of renal artery branching patterns according to Kang et al. show eight different types based on peri-hilar and segmental variations. Type Ia features a single main renal artery bifurcating into two branches. Type Ib shows a single main renal artery trifurcating into three branches, while Type Ic demonstrates quadrifurcation into four branches. Type IIa has two main renal arteries each bifurcating into two branches, and Type IIb features two main renal arteries each trifurcating into three branches. Type IIc presents two main renal arteries, each quadrifurcating into four branches. In Type IIIb, three main renal arteries each bifurcate into two branches, whereas Type IIIc has three main renal arteries each trifurcating into three branches. Permission to use this figure has been granted (65).

Another classification of branching patterns is segmental arteries, from which each segment arises. In 1954, Graves observed and classified the variations in segmental arteries. These classifications and groupings are still used as the basis of many studies. Graves stated that the apical segmental artery has four types of variations (62). Type I lesions arise in the upper segmental artery. Type II arises from the junction between the anterior and posterior divisions of the main-stem renal artery. Type III arises at the junction of the main renal artery with the aorta, and enters the apical segment outside the hilum. Type IV arises from posterior division. Graves divided the branching of the upper, middle, and lower segmental arteries into three groups. Group I described the lower segmental artery arising first, with the upper and middle segments sharing a common origin. Group II described the upper segmental artery arising first, with the middle and lower arteries sharing a common origin. Group III includes the upper, middle, and lower segmental arteries arising from a common origin (62). As a follow-up to Graves' work, Fine and Keen introduced the concept of primary and secondary branches of renal arteries (63). Their findings state that the renal artery typically divides into three primary branches: upper, lower, and posterior, with the intermediate and middle branches recognized as secondary. This division can be variable, in which the branch arises first in the renal artery. While discussing segmental branching, it is important to note that these variants are often accompanied by perihilar branching. As such, the nomenclature of these variations is not definitive and vast in the literature. Although Graves, Fine, and Keen are responsible for their original classifications, many studies have created classifications to suit their own parameters. A great example of this is Kang et al.'s study of 64 MDCT angiograms. In this study, as shown in Figure 2, eight different branching patterns were defined using a combination of perihilar and segmental variations (64). Owing to the lack of universal definitions in the literature, the overall incidence of segmental branching is discrepant. Nevertheless, surgeons should remember that segmental branching is common and should be carefully observed on a case-by-case basis (30, 65, 66).

##### 3.1.2.3 Multiple renal arteries

**Figure 3:**
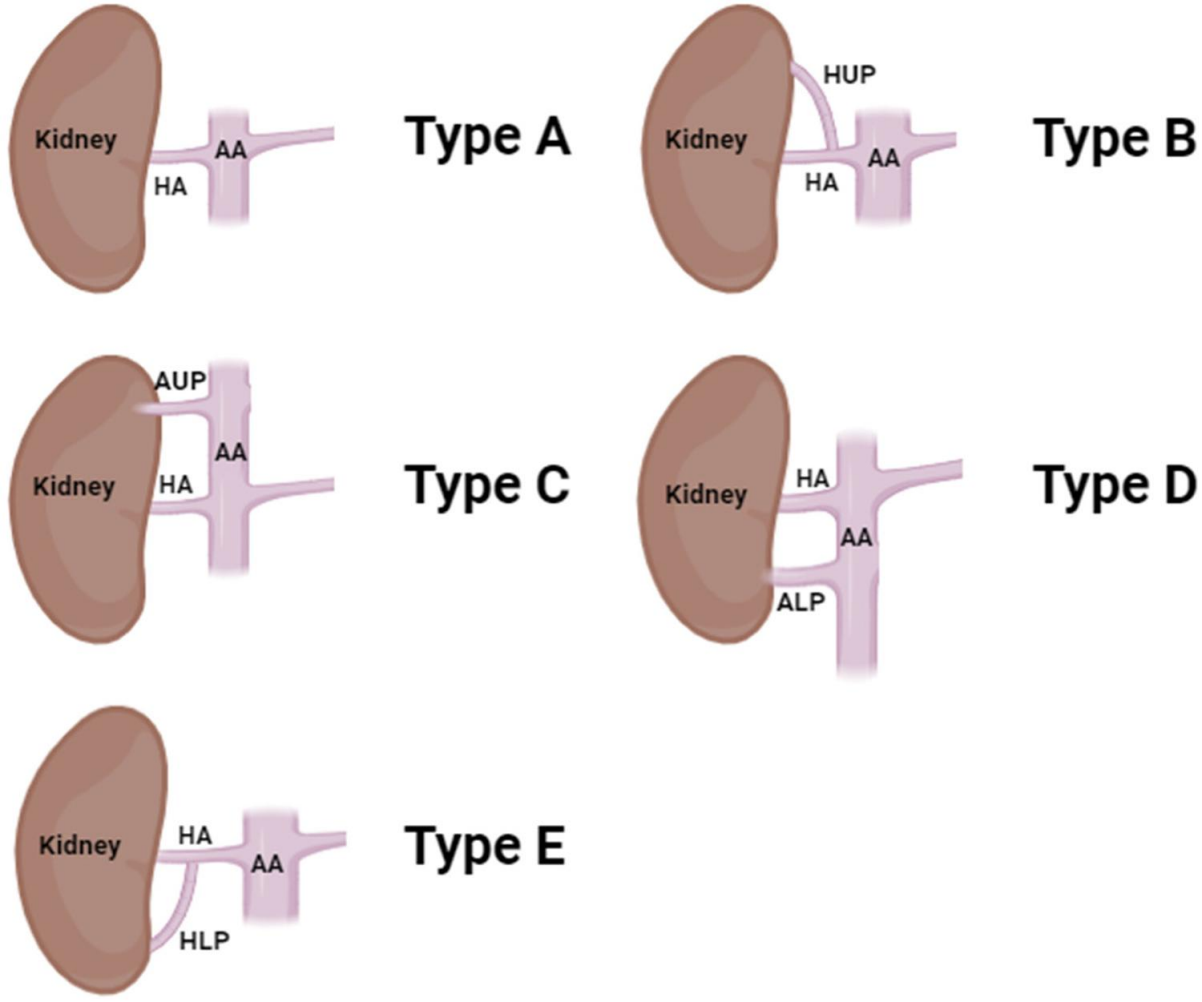
Illustration of the five subcategories of accessory renal arteries based on position. Type A: Accessory artery arises directly from the abdominal aorta and supplies the kidney. Type B: Artery arises from the aorta and supplies the upper pole with a hilar upper polar artery (HUP). Type C: Artery arises from the aorta and supplies the upper pole with an aortic upper polar artery (AUP). Type D: Artery arises from the aorta and supplies the lower pole with an aortic lower polar artery (ALP). Type E: Artery arises from the aorta and supplies the lower pole with a hilar lower polar artery (HLP). AA, abdominal aorta; K, kidney; ALP, aortic lower polar artery; AUP, aortic upper polar artery; HA, hilar artery; HLP, hilar lower polar artery; HUP, hilar upper polar artery, modified from (69).

The most common arterial variation is the renal multiplicity. Similar to peri-hilar branching variations, one explanation for arterial multiplicities is the failure of lateral splanchnic arteries to degenerate during embryo formation. Approximately 30% of the population has more than one renal artery (34). Although this is an overall incidence, many studies have reported a wide range of distributions. In a study of 1,859 donor nephrectomy candidates, multiple arteries were present in 18.3% and 22.6% of patients on the right and left sides, respectively (57). Arterial multiplicities can be unilateral or bilateral; however, unilateral presentations are more prevalent. In a study of 403 kidney donors, multiple renal arteries occurred bilaterally in 10.2% of donors and unilaterally in 20.8% (67). When discussing renal arterial multiplicity, it is important to clarify the difference between additional renal arteries and accessory renal arteries because terminology surrounding these vessels differs in the literature. An extrarenal artery is defined as an artery other than the main renal artery arising from the aorta and terminating at the hilum of the kidney. An accessory renal artery, also known as an aberrant artery, arises from aortic branches. The accessory renal arteries enter the kidneys as polar arteries and connect to the upper or lower poles. Individuals may have one, two, three, or four accessory arteries. There are five subcategories of accessory renal arteries based on their position: aortic hilar, renal upper polar, aortic upper polar, aortic lower polar, and renal lower polar (68). These subcategories are illustrated in Figure 3. The nomenclature of “hilar” and “polar” describes the area of the kidney where the arteries terminate in the kidney. Polar arteries may have an aortic or renal origin. In one study of 60 cadaver kidneys and 583 abdominal CT images, these five types were identified and recorded. Aortic hilar arteries have a 79% incidence in cadavers and a 95% incidence in patients, making it the most prevalent variation. Renal upper polar arteries were present in 10% of cadavers and 2% of patients. Aortic upper polar arteries have an incidence of 5% in cadavers and 2% in patients. The incidence of the aortic lower polar artery was 3% in cadavers and 1% in patients. Renal lower polar arteries were present in 2% of cadavers and in <0.1% of patients. The same study also reported the number of arteries that reached one kidney. One artery was found in 78% of cadavers and 88% of patients. Two were present in 19% of the cadavers and 12% of the patients. Three arteries were discovered in 2% of cadavers; however, this variation was not observed in patients. Finally, 2% of cadavers had four arteries, but this variation was not observed in patients (69). This study found no significant difference between sexes when considering the incidence of these variations. However, previous studies have cited sex and race differences (57, 70). Specific investigations into the prevalence of multiple arteries regarding sex and ethnic differences would be insightful in the future as operations on multiple renal arteries become more accepted.

#### 3.1.3 Venous variations

##### 3.1.3.1 Overview of venous variation

Renal venous variations are also common and are particularly relevant on the left side, which is usually preferred for donation. Variations in tributaries of the renal vein are expected, especially in the left renal vein. The right renal vein does not frequently have major tributaries, but may occasionally drain adrenal, gonadal, or retroperitoneal veins. By contrast, these vessels commonly join the left renal vein and may show variable patterns of communication. One meta-analysis reported that communicating veins between the left renal vein and retroperitoneal veins occur in 34%–75.8% of clinical reports (71). Because venous drainage patterns may affect hilar dissection, control of collateral veins, and back-table planning, these variants are clinically significant even when they do not independently worsen long-term graft outcomes.

##### 3.1.3.2 Retroaortic and circumaortic left renal veins

Retroaortic and circumaortic left renal veins are among the most clinically relevant venous variants in transplantation (71–73). A retroaortic left renal vein passes posterior to the aorta and has been classified into four types: orthotopic, oblique, circumaortic, and a vein joining the left common iliac vein (74–76). In type I, the vein passes posterior to the aorta at approximately the same vertebral level as a normal left renal vein. In type II, the vein passes obliquely behind the aorta and joins the gonadal and ascending lumbar veins before entering the inferior vena cava above the common iliac confluence. In type III, or circumaortic variation, anterior and posterior venous channels encircle the aorta. In type IV, the retroaortic vein joins the left common iliac vein (76).

**Figure 4:**
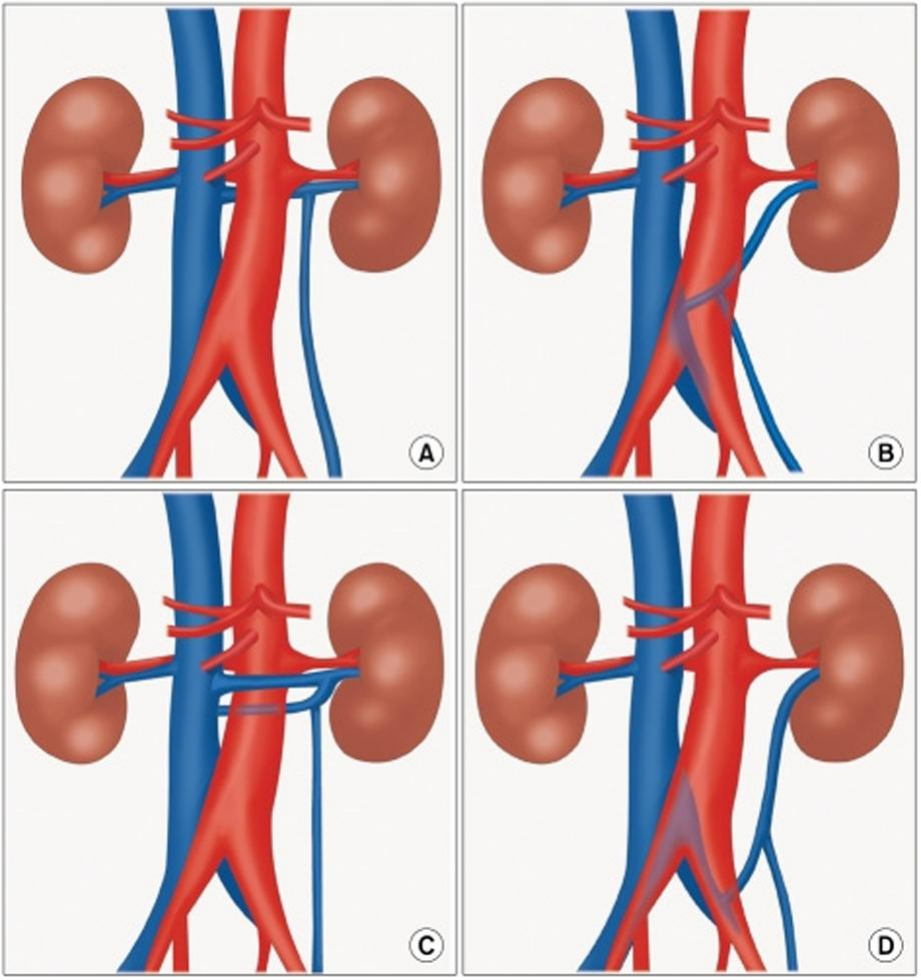
Illustration of the four types of retroaortic left renal vein variations. This figure illustrates the four types of retroaortic left renal veins. Image **(A)** represents Type I, orthotropic, where the renal vein follows a standard pathway behind the aorta. Image **(B)** shows Type II, oblique, where the renal vein crosses the aorta at an oblique angle. Image **(C)** depicts Type III, circumaortic, where the renal vein wraps around the aorta, forming a ring-like structure. Image **(D)** illustrates Type IV, where the renal vein joins the common iliac vein directly. Permission to use this figure has been granted (76).

Embryologically, these patterns are thought to result from persistence or regression of different portions of the ventral and dorsal renal venous system (77–79). In an MDCT angiography study of 1,856 patients, retroaortic variants were identified in 68 patients (3.6%), including type I in 1.4%, type II in 1.2%, type III in 0.9%, and type IV in 0.2% (80). In another study of 1,452 patients undergoing MDCT angiography, 2.1% had a retroaortic vein and 2.1% had a circumaortic vein (81). These figures are broadly consistent with previous literature suggesting an overall incidence of approximately 3% (74) (Figure 4), although variation between studies remains considerable. Available studies have not demonstrated consistent associations with sex or race, but the evidence remains limited.

##### 3.1.3.3 Multiple renal veins

Venous multiplicity is the most common renal venous variation. It occurs in approximately 16% of the population (82), most often as two veins on the same side. As with arterial multiplicity, it may occur unilaterally or bilaterally, although it is considerably more common on the right than on the left. A meta-analysis reported right-sided multiplicity in 14.3%–19.2% of patients, compared with 1.3%–3.2% on the left (74). One explanation for this asymmetry is the more complex embryogenesis of the left side, which may reduce persistence of additional venous channels (83). In a cadaveric cohort of 119 subjects, 18% had two right renal veins and 5% had three, whereas on the left, 5.9% had two veins and only 0.8% had three (84).

Although right-sided venous multiplicity is more common and the left kidney is typically favoured for living donation, transplant surgeons should still be aware of this variation and its implications for hilar dissection, venous reconstruction, and recipient implantation.

Overall, the literature suggests that peri-hilar and segmental arterial branching, multiple renal arteries, retroaortic or circumaortic left renal veins, and multiple renal veins are the variants most likely to be encountered in live donor nephrectomy. These variants are therefore of greatest relevance to preoperative imaging, donor selection, and operative planning.

## 4 Clinical implications for kidney transplantation

### 4.1 Impact of vascular variation on transplant outcomes

The impact of renal vascular variation on transplant outcomes has been studied extensively in both living and deceased donor transplantation. Vascular variants such as multiple renal arteries and complexity of venous anatomy may increase the complexity of the operative procedure and the requirement for reconstruction. However, the reported effect on graft survival and postoperative complications has been inconsistent in the literature. The literature has shown inconsistent findings regarding differences in surgical era, donor type, imaging techniques, and operative approach. Selected key studies examining the relationship between renal vascular variations and transplant-related outcomes are summarised in the following Table 2.

**Table 2:** Clinical studies evaluating renal vascular variations in kidney transplantation.

Study	Study design	Variant studied	Main findings	Limitations
Hsu et al.	Retrospective cohort	Multiple renal arteries	Increased operative time but similar long-term graft survival	Single-center study
Ashraf et al.	Retrospective review	Multiple arteries	Higher early complication rates	Limited follow-up
Paramesh et al.	20-year review	Multiple vessels	Comparable graft and patient survival	Heterogeneous surgical techniques
Hos˘tiuc et al.	Meta-analysis	Renal vein variants	Venous variants generally manageable	Variability between included studies

#### 4.1.1 Arterial variants and outcomes

Variant arterial anatomy has traditionally been associated with greater technical complexity and higher perioperative risk. Multiple renal arteries have been linked to acute rejection, longer warm ischemia time, vascular thrombosis, tubular necrosis, and hemorrhagic complications (48, 85, 86). More recent data continue to show that multiple renal arteries may be associated with longer operative and ischemia times, higher complication rates, and delayed graft function (87, 88). One study reported more recipient vascular and ureteral complications, although no differences were found in blood loss, donor hospital stay, or donor surgical complications (88). This suggests that the principal burden of arterial complexity may fall more on recipient-side outcomes and early graft function than on donor morbidity.

At the same time, the literature increasingly indicates that these short-term technical challenges do not necessarily translate into worse long-term results. Long-term allograft and patient survival have generally remained comparable between grafts with single arteries and those with multiple arteries (20, 88–90). A 20-year cross-sectional review of 2,674 live donor transplant patients from 1997 to 2017 found no significant differences in patient survival, graft survival, or long-term health outcomes between single-vessel and multiple-vessel grafts (91).

Taken together, these findings suggest that the effect of arterial multiplicity should be interpreted in the context of modern surgical practice. Earlier reports of arterial stenosis and thrombosis often included deceased donors and open nephrectomies (92, 93), whereas studies restricted to laparoscopic donor nephrectomy have not demonstrated the same degree of risk (94, 95). This distinction is important in critically appraising the literature, as older data may overestimate the hazards of arterial variation in contemporary live donor transplantation. Nevertheless, heterogeneity in study design and terminology remains a limitation, and further studies focused specifically on modern laparoscopic series would strengthen the evidence base.

#### 4.1.2 Venous variants and outcomes

Retroaortic renal veins have been associated in non-transplant settings with haematuria, flank pain, varicocele, and abdominal pain (72), and in retroperitoneal surgery they have been considered a potential source of bleeding risk (73, 74). However, these reports are not specific to laparoscopic donor nephrectomy. In the transplant literature, although retroaortic venous anatomy may increase technical difficulty during nephrectomy, it has not been shown to adversely affect graft or patient outcomes. Studies have reported no significant differences in operative time, blood loss, warm ischaemia time, donor hospital stay, complications, or transplant function when compared with standard venous anatomy (75, 76). Cold ischaemia and revascularisation times have also been reported as comparable (78).

Similar conclusions have been drawn for venous multiplicity. No significant differences have been found in blood loss, thrombosis, length of stay, 30-day readmission, or rejection rates when compared with singular venous anatomy (96). Overall, the literature suggests that venous variants are generally manageable and may carry less adverse impact on outcomes than arterial variants. Their importance therefore lies primarily in surgical planning and technical execution rather than in long-term graft survival.

### 4.2 Preoperative identification and imaging assessment

#### 4.2.1 Imaging modalities

**Figure 5:**
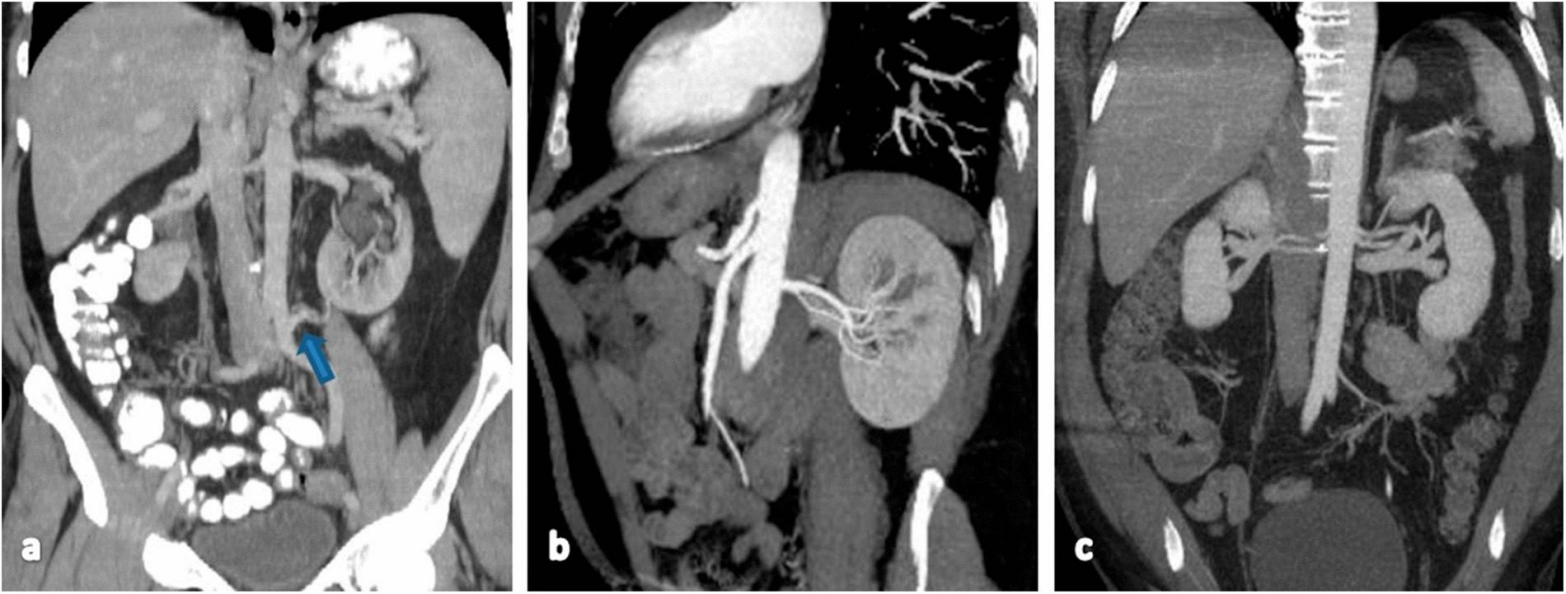
Computed tomography (CT) images of renal vascular variations. This figure displays computed tomography images highlighting different renal vascular variations. Image **(a)** shows a left inferior accessory polar artery. Image **(b)** depicts early branching of the left renal artery. Image **(c)** illustrates bilateral hilar accessory arteries. These variations are important considerations in renal surgery and transplantation planning. Permission to use this figure has been granted (98).

**Figure 6:**
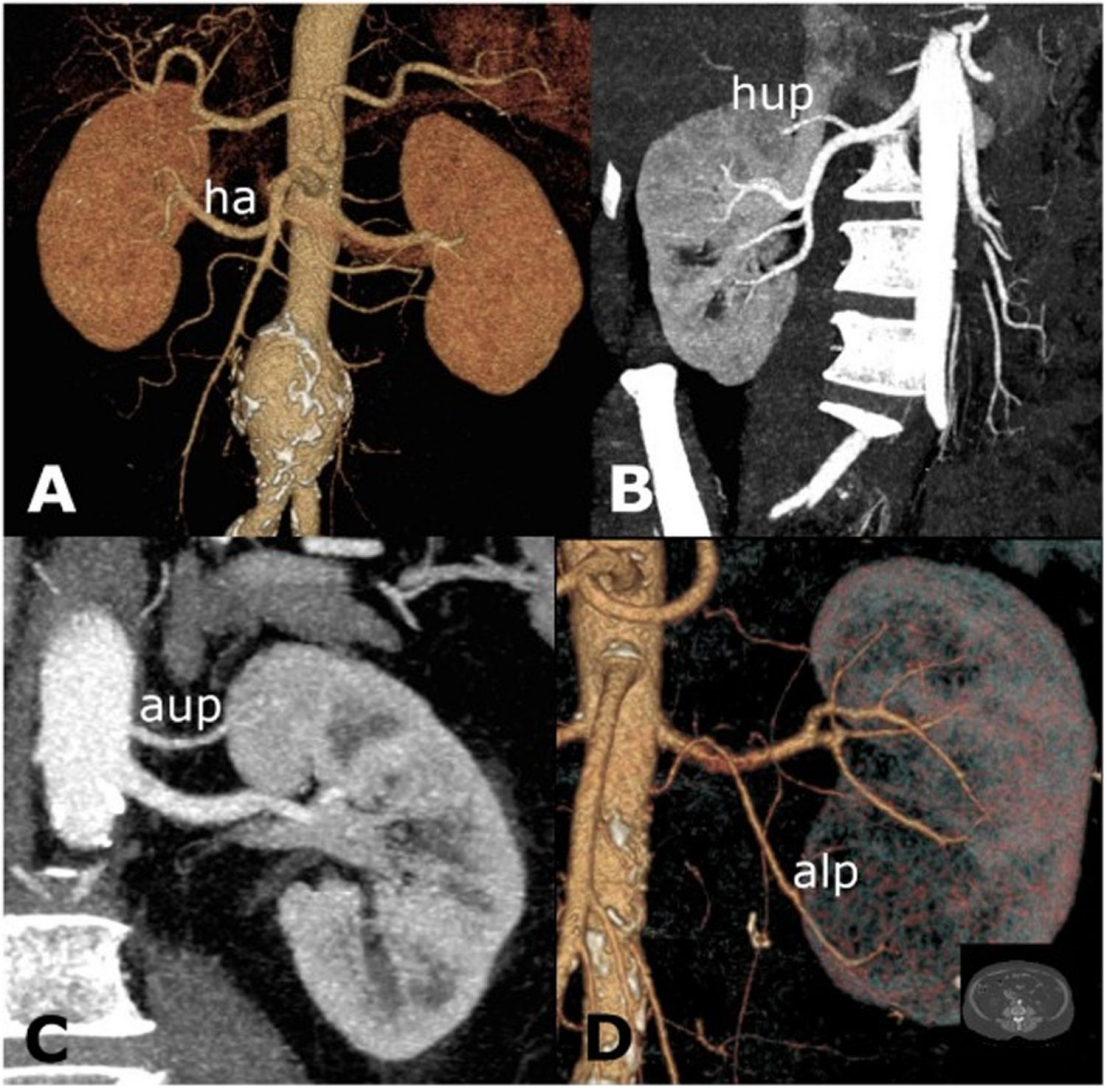
Computed tomography (CT) imaging of accessory artery subtypes based on position. This figure presents CT images illustrating various subtypes of accessory arteries based on their position. Image **(A)** is a volume rendering CT showcasing a hilar artery. Image **(B)** displays a hilar upper polar artery. Image **(C)** shows an aortic upper polar artery. Image **(D)** is a volume rendering CT depicting an aortic lower polar artery. These variations are crucial for pre-surgical planning and management of renal vascular conditions. Permission to use this figure has been granted (66).

Accurate preoperative assessment is central to the management of renal vascular variation in transplantation. Reported imaging methods include computed tomography angiography (CTA), multidetector computed tomography angiography (MDCTA), magnetic resonance angiography (MRA), and, more broadly, CT and MRI-based techniques (96–98). In transplant practice, angiographic assessment is particularly important because it provides detailed vascular mapping relevant to donor selection and operative planning. CTA remains the most commonly used method for preoperative vascular assessment (Figure 5). It provides cross-sectional imaging with contrast enhancement and allows identification of renal arterial and venous anatomy, including multiplicity, branching patterns, and hilar relationships. MDCTA offers additional advantages, including faster image acquisition, greater patient coverage, higher resolution, and volume-rendered data in addition to axial slices (Figure 6) (99). These features make it particularly useful when vascular detail is critical. MRA may be preferred in patients who cannot undergo iodinated contrast studies or in whom radiation exposure is undesirable, although small vessels may be less well visualised and separate arterial and venous definition may be less precise than with CTA (100–102). Although magnetic resonance angiography avoids ionising radiation, gadolinium-based contrast agents should be used cautiously in patients with advanced renal impairment because of the potential risk of nephrogenic systemic fibrosis. Emerging technologies such as three-dimensional (3D) vascular reconstruction, 3D printing, and artificial intelligence-assisted vascular mapping may further improve preoperative planning and surgical simulation in complex renal vascular anatomy, although current evidence remains limited (97).

#### 4.2.2 Strengths and limitations of imaging

Although preoperative imaging is essential, it is not infallible. In one MDCT study of 97 donors, 21 accessory renal arteries were identified, but two additional arteries were only recognised after surgery, giving a detection rate of 91.3% (99). Similarly, in a study of 288 kidney donations, MRI failed to predict arterial anatomy in 23 of 220 donor studies, while angiography failed in 3 of 101 cases (73). These findings highlight an important limitation of the literature and of clinical practice: even modern imaging may underestimate vascular complexity. Surgeons should therefore remain prepared to encounter unanticipated vessels intraoperatively.

The choice of modality also reflects practical considerations. CTA and MDCTA are generally favoured because of speed, availability, and excellent visualisation of small vessels, whereas MRA avoids radiation but may be more expensive, less available, and less precise for small vascular structures. This is particularly relevant in a transplant setting, where small accessory arteries and short pedicles may have major operative consequences (Table 3).

**Table 3:** Comparison of vascular imaging modalities.

Modality	Advantages	Disadvantages
CT/CTA	Use of “slice” images; lower cost; shorter exam times; widely available; can accommodate larger patients; excellent visualisation of small vessels	Radiation; iodine contrast agent
MDCT/MDCTA	Faster imaging acquisition speed; more coverage; higher resolution; volume data in addition to slice data	Radiation; iodine contrast agent; not as widely available as traditional CT due to higher cost of machine; larger data requires more time to assess
MRI/MRA	No radiation; gadolinium-based contrast agent; great visualisation of soft tissues	Expensive; medical devices can interfere; smaller machines; individual images of vessels may not be possible

When variant anatomy is identified, imaging should define the features most relevant to operative planning. These include renal location, length, axis, vessel number, type, origin, diameter, inter-vessel distance, and pedicle length (100, 101). Arterial pedicle length is measured from the ostium to the first segmental branch and should be sufficient to allow safe anastomosis; one study suggested that lengths less than 26 mm on the right and 16 mm on the left may increase the likelihood of requiring multiple anastomoses (102, 103). Arterial luminal diameter should also be assessed, with a minimum of 10 mm generally considered necessary for safe anastomosis (102).

Early branching is particularly important because branches arising within 15–20 mm of the aortic ostium may limit the length available for clamping and reconstruction (103, 104). On the right side, the relationship of the first segmental branch to the inferior vena cava is especially relevant because the artery passes posterior to the IVC and may be difficult to expose laparoscopically (102). Where multiple arteries are present, interarterial distance should be noted, since closely situated arteries may allow single reconstruction whereas widely separated arteries may require separate anastomoses (105).

Similar principles apply to venous anatomy. Venous pedicle length and luminal diameter should be assessed, and all draining veins greater than 5 mm should be reported, particularly posterior lumbar or gonadal tributaries that may be difficult to visualise laparoscopically (102, 106). In circumaortic or duplicated left renal veins, comparative diameters of the venous channels are important because a small branch contributing less than 20% of total drainage may be considered for ligation (107). Imaging should also identify the renal pelvis and ureteropelvic junction where relevant, particularly when vascular anatomy may contribute to obstruction or complicate hilar dissection (102).

Accurate interpretation of these findings requires multidisciplinary collaboration among transplant surgeons, radiologists, nephrologists, and urologists. This collaborative approach improves donor selection, surgical planning, and perioperative safety.

### 4.3 Surgical planning and management strategies

#### 4.3.1 Management of arterial multiplicity and branching variants

Variant anatomy may complicate organ retrieval and implantation, particularly in laparoscopic donor nephrectomy where intraoperative visualisation is limited (108). Multiple arteries are of particular concern because the renal arterial supply consists of end arteries, making preservation of perfusion critical. When arteries are close together near the aortic origin, they may be stapled together with the main renal artery during retrieval. When interarterial distance prevents this, they may need to be clipped or stapled separately (105). Small polar arteries may escape preoperative detection and only become apparent during retrieval. These vessels, especially lower polar arteries supplying the ureter, require careful preservation where possible (109–111) Some authors suggest that very small upper polar arteries supplying less than 10% of the renal parenchyma or measuring less than 2 mm may be ligated when safe reconstruction is unlikely (112–114). Although this may risk focal infarction of the upper pole (56). These decisions remain case-specific and depend on anatomy, perfusion territory, and surgical judgement.

Early branching variants may also be challenging because they shorten the usable arterial stump. In such cases, meticulous dissection is required to preserve enough length for reconstruction and anastomosis. Because peri-hilar and segmental patterns are heterogeneous, no universal operative approach applies, and preoperative imaging must guide case-by-case planning.

Following donor retrieval, bench microsurgery may be required to create a configuration suitable for recipient implantation. When two renal arteries are present, the vessels may be spatulated to create a common trunk and permit a single arterial anastomosis, using either end-to-side or side-to-side techniques (105, 115). The side-to-side configuration is commonly referred to as the “pants technique” (116–118). In selected cases, the recipient's iliac artery or saphenous vein may be used to facilitate reconstruction and create a common channel. If this is not feasible because of size discrepancy or interarterial distance, separate arterial anastomoses may be required (105). Smaller accessory arteries may be anastomosed end-to-side to a larger artery in a parachuting technique, which may reduce warm ischaemia time and anastomotic complications (119). Even in kidneys with three or more arteries, successful construction of a single orifice has been reported using side-to-side techniques (120, 121).

During implantation, surgeons may choose the external iliac artery for end-to-side anastomosis or the internal iliac artery for end-to-end anastomosis. The external iliac artery is often preferred because of its superficial location and larger calibre (122), whereas the internal iliac artery may be selected when the external iliac artery is diseased or damaged (115). The drawbacks of this artery include its smaller size, potential erectile dysfunction resulting from penile vascular insufficiency, and the need to ligate branches to facilitate end-to-end anastomosis with the donor renal artery (123, 124). Ultimately, this decision is the operating surgeon's preference based on circumstance, as either artery can be utilized, and both techniques report similar short- and long-term outcomes (125). Following anastomosis, revascularization of the graft occurs. In case of multiple anastomoses, revascularization can be performed either simultaneously with the entire graft or sequentially. Sequential revascularization is preferred, allowing the kidney to begin reperfusion while the surgeon operates on other anastomoses. This strategy reduces the warm ischemia time and the incidence of acute tubular necrosis (109). The main renal artery was first anastomosed. Vascular clamps are then released, and the kidney is partially revascularized until the other accessory artery is successfully anastomosed and total renal perfusion is sustained (42).

#### 4.3.2 Management of venous variants

Management strategies for venous variation are generally simpler than for arterial variation but remain important. In double renal veins of unequal size, the larger vein is usually sufficient for drainage and the smaller vein may be ligated without consequence (126). If the two veins are roughly equal in size, bench surgery of the kidney may be required to create a common trunk for anastomosis using the same techniques as arterial trunk formation. Alternatively, both veins could be anastomosed separately to the external iliac vein (112). Singular anastomosis, achieved either through ligation or bench surgery of the vessels, is preferable for avoiding surgical complications (127). However, if multiple anastomoses are necessary, a sequential vascularization technique can be used (128). The renal vein entered the hilum inferiorly in the orthotropic and oblique retroaortic veins, making the kidney significantly less mobile. As a result, this variant is often accompanied by other anatomical variations in the venous tributaries, including the lumbar, adrenal, and gonadal veins. In one study of 126 potential donors, three of ten cases with a circumaortic left renal vein had a lumbar or gonadal vein larger than 5 mm draining into either the preaortic or retroaortic branch. In the same study, one donor had both a 5 mm lumbar vein and a 5 mm gonadal vein connecting to the retroaortic branch (106). Small collateral veins contributing to the left renal vein may need to be ligated. The retroaortic veins create a wider gap between the renal arteries and veins. As such, the operative technique involves dissection along the vein's lateral aortic border and shortening the total length, which still exceeds the length of the right renal vein (129). For circumaortic veins, the retroaortic portion is typically smaller and can be safely sacrificed. In contrast, the preaortic portion is clipped at the opening of the inferior vena cava (130).

## 5 Limitations of the current literature and future directions

As a narrative review, it is inherently susceptible to selection bias and does not provide the methodological rigor of a systematic review or meta-analysis. Although multiple databases were searched, no formal quality assessment of included studies was performed.

Moreover, only English-language studies were included, which may have left out important international data. There is significant heterogeneity throughout the literature relative to study design, imaging methodologies, surgical methods, and terminology for discussion of renal vascular variations that precludes direct comparison between studies. Furthermore, some older studies included open nephrectomy and deceased donor transplantation, which may not fully reflect outcomes in contemporary laparoscopic transplant practice. Even with these limitations, the present review offers a targeted clinical synthesis of the available evidence of renal vascular.

## 6 Conclusion

Vascular variations of the kidney are frequent and are often encountered in kidney transplantation. The most clinically important variants are peri-hilar and segmental branching of the arteries, multiple renal arteries, retroaortic or circumaortic left renal vein and multiple renal veins. These variants may increase the operative complexity, the requirements for reconstruction, the warm ischaemia time and the risk of early postoperative complications, especially in recipients requiring multiple vascular anastomoses.

However, current evidence suggests that with careful donor selection, accurate preoperative vascular imaging, multidisciplinary planning and modern reconstructive techniques, favourable long-term graft and patient outcomes can still be achieved. Vascular variations can represent a great technical challenge for donor nephrectomy and recipient implantation but should not be considered as absolute contraindications to transplantation in carefully selected patients.

The literature also suggests that some complications historically attributed to vascular variation may have been overestimated in previous studies of deceased donors and open nephrectomy techniques. In modern transplant practice, advances in vascular reconstruction, laparoscopic donor nephrectomy and improved imaging modalities have permitted the safe use of anatomically complex grafts. However, continued standardisation of terminology, imaging assessment and outcome reporting is required to further improve the evidence base and optimise donor utilisation and transplant outcomes.

## References

[1] Levey AS Eckardt KU Tsukamoto Y Levin A Coresh J Rossert J. Definition and classification of chronic kidney disease: a position statement from kidney disease: improving global outcomes (KDIGO). Kidney Int. (2005) 67(6):2089–100. 10.1111/j.1523-1755.2005.00365.x 15882252

[2] Kazancioglu R. Risk factors for chronic kidney disease: an update. Kidney Int Suppl. (2013) 3(4):368–71. 10.1038/kisup.2013.79 PMC4089662 25019021

[3] Levey AS Becker C Inker LA. Glomerular filtration rate and albuminuria for detection and staging of acute and chronic kidney disease in adults. J Am Med Assoc. (2015) 313(8):837. 10.1001/jama.2015.0602 PMC4410363 25710660

[4] Taal MW Chertow GM Marsden PA Skorecki K Alan S Brenner BM. Brenner and Rector’s the Kidney E-Book. Elsevier Health Sciences (2011).

[5] Wouk N. End-stage renal disease: medical management. Am Fam Physician. (2021) 104(5):493–9. 34783494

[6] Kovesdy CP. Epidemiology of chronic kidney disease: an update 2022. Kidney Int Suppl. (2022) 12(1):7–11. 10.1016/j.kisu.2021.11.003 PMC9073222 35529086

[7] Lv JC Zhang LX. Prevalence and disease burden of chronic kidney disease. Adv Exp Med Biol. (2019) 1165:3–15. 10.1007/978-981-13-8871-2_1 31399958

[8] Hill NR Fatoba ST Oke JL Hirst JA O'Callaghan CA Lasserson DS. Global prevalence of chronic kidney disease - a systematic review and meta-analysis. PLoS One. (2016) 11(7):e0158765. 10.1371/journal.pone.0158765 27383068 PMC4934905

[9] Jha V Garcia-Garcia G Iseki K Li Z Naicker S Plattner B. Chronic kidney disease: global dimension and perspectives. Lancet. (2013) 382(9888):260–72. 10.1016/S0140-6736(13)60687-X 23727169

[10] McCullough KP Morgenstern H Saran R Herman WH Robinson BM. Projecting ESRD incidence and prevalence in the United States through 2030. J Am Soc Nephrol. (2019) 30(1):127–35. 10.1681/ASN.2018050531 30559143 PMC6317596

[11] Liyanage T Ninomiya T Jha V Neal B Patrice HM Okpechi I. Worldwide access to treatment for end-stage kidney disease: a systematic review. Lancet. (2015) 385(9981):1975–82. 10.1016/S0140-6736(14)61601-9 25777665

[12] Marroquin CE. Patient selection for kidney transplant. Surg Clin North Am. (2019) 99(1):1–35. 10.1016/j.suc.2018.09.002 30471735

[13] Scandling JD. Kidney transplant candidate evaluation. Semin Dial. (2005) 18(6):487–94. 10.1111/j.1525-139X.2005.00094.x 16398711

[14] Standring S Ellis H Healy J Johnson D Williams A Collins P. Gray’s anatomy: the anatomical basis of clinical practice. Am J Neuroradiol. (2005) 26(10):2703.

[15] Wadei HM Heckman MG Rawal B Taner CB Farahat W Nur L. Comparison of kidney function between donation after cardiac death and donation after brain death kidney transplantation. Transplantation. (2013) 96(3):274–81. 10.1097/TP.0b013e31829807d1 23778649

[16] Abramowicz D Cochat P Claas FH Heemann U Pascual J Dudley C. European renal best practice guideline on kidney donor and recipient evaluation and perioperative care. Nephrol Dial Transplant. (2015) 30(11):1790–7. 10.1093/ndt/gfu216 25007790

[17] Andrews PA Burnapp L Manas D Bradley JA Dudley C. Summary of the British transplantation society/renal association UK guidelines for living donor kidney transplantation. Transplantation. (2012) 93(7):666–73. 10.1097/TP.0b013e318247a7b7 22456484

[18] Ahmadi AR Lafranca JA Claessens LA Imamdi RMS Ijzermans JNM Betjes MGH. Shifting paradigms in eligibility criteria for live kidney donation: a systematic review. Kidney Int. (2015) 87(1):31–45. 10.1038/ki.2014.118 24786706

[19] Roll GR Cooper M Verbesey J Veale JL Ronin M Irish W. Risk aversion in the use of complex kidneys in paired exchange programs: opportunities for even more transplants? Am J Transplant. (2022) 22(7):1893–900. 10.1111/ajt.17008 35181991 PMC9543328

[20] Bruyere F Doumerc N. Robotic kidney transplantation: dream or future? Curr Opin Urol. (2018) 28(2):139–42. 10.1097/MOU.0000000000000476 29303915

[21] Khalil A Mujtaba MA Taber TE Yaqub MS Goggins W Powelson J. Trends and outcomes in right vs. left living donor nephrectomy: an analysis of the OPTN/UNOS database of donor and recipient outcomes–should we be doing more right-sided nephrectomies? Clin Transplant. (2016) 30(2):145–53. 10.1111/ctr.12668 26589133

[22] Tennankore KK Kim SJ Alwayn IPJ Kiberd BA. Prolonged warm ischemia time is associated with graft failure and mortality after kidney transplantation. Kidney Int. (2016) 89(3):648–58. 10.1016/j.kint.2015.09.002 26880458

[23] Aydin C Berber I Altaca G Yigit B Titiz I. The outcome of kidney transplants with multiple renal arteries. BMC Surg. (2004) 4:4. 10.1186/1471-2482-4-4 15018624 PMC362880

[24] Dalal R Bruss ZS Sehdev JS. Physiology, renal blood flow and filtration. In: StatPearls. Treasure Island (FL): StatPearls Publishing (2023). https://www.ncbi.nlm.nih.gov/books/NBK482248 29489242

[25] Alamilla-Sanchez M Alcalá Salgado MA Ulloa Galván VM Yanez Salguero V Yamá Estrella MB Morales López EF. Understanding renal tubular function: key mechanisms, clinical relevance, and comprehensive urine assessment. Pathophysiology. (2025) 32(3):33. 10.3390/pathophysiology32030033 40700075 PMC12285995

[26] Ponticelli CE. The impact of cold ischemia time on renal transplant outcome. Kidney Int. (2015) 87(2):272–5. 10.1038/ki.2014.359 25635719

[27] Singhal SK Alzaki A Khati NJ Boumezrag M Razjouyan F Venbrux AC. Chapter 30: Renal vasculature. In: Mauro MA Murphy KP Thomson KR Venbrux AC Morgan RA, editors. Image-Guided Interventions, 3rd ed. Boston: Elsevier (2020). p. 255–62.

[28] Hall JE Hall ME. Guyton and Hall Textbook of Medical Physiology. 14th ed Philadelphia: Elsevier (2020).

[29] De Groote R Sundaram C De Backer P. Renal anatomy, physiology, and its clinical relevance to renal surgery. In: Wiklund P Mottrie A Gundeti MS Patel V, editors. Robotic Urologic Surgery. Cham: Springer International Publishing (2022). p. 407–20.

[30] Rani N Singh S Dhar P Kumar R. Surgical importance of arterial segments of human kidneys: an angiography and corrosion cast study. J Clin Diagn Res. (2014) 8(3):1–3. 10.7860/JCDR/2014/7396.4086 24783063 PMC4003595

[31] Feher J. Chapter 7.2: Functional anatomy of the kidneys and overview of kidney function. In: Feher J, editor. Quantitative Human Physiology, 2nd ed. Boston: Academic Press (2017). p. 698–704.

[32] Partin AW Dmochowski RR Kavoussi LR Peters C. Campbell-Walsh-Wein Urology. 12th ed. Philadelphia, PA: Elsevier (2021).

[33] Kaufman DP Basit H Knohl SJ. Physiology, glomerular filtration rate. In: StatPearls. Treasure Island (FL): StatPearls Publishing (2023); Copyright © 2023, StatPearls Publishing LLC. 29763208

[34] Standring S. Gray’s Anatomy: The Anatomical Basis of Clinical Practice. 41st ed. Philadelphia: Elsevier Churchill Livingstone (2015).

[35] Yu ASL Chertow GM Luyckx VA Marsden PA Skorecki K Taal MW. Brenner & Rector’s the Kidney. 11th ed. Philadelphia, PA: Elsevier (2020).

[36] Madrazo-Ibarra A Vaitla P. Histology, nephron. In: StatPearls. Treasure Island (FL): StatPearls Publishing (2023); Copyright © 2023, StatPearls Publishing LLC. 32119298

[37] Rayner HC Thomas ME Milford DV. Kidney anatomy and physiology: the basis of clinical nephrology. In: Rayner HC Thomas ME Milford DV, editors. Understanding Kidney Diseases. Cham: Springer International Publishing (2020). p. 1–9.

[38] Rayner HC Thomas M Milford D. Kidney Anatomy and Physiology. Switzerland: Springer International Publishing AG (2015).

[39] Tucker WD Shrestha R Burns B. Anatomy, abdomen and pelvis: inferior vena cava. In: StatPearls. Treasure Island (FL): StatPearls Publishing (2023); Copyright © 2023, StatPearls Publishing LLC. 29493975

[40] Bowdino CS Owens J Shaw PM. Anatomy, abdomen and pelvis, renal veins. In: StatPearls. Treasure Island (FL): StatPearls Publishing (2023); Copyright © 2023, StatPearls Publishing LLC. 30855882

[41] Wood D Greenwell T. Surgical anatomy of the kidney and ureters. Surgery. (2013) 31(7):326–8. 10.1016/j.mpsur.2013.04.015

[42] Mahadevan V. Anatomy of the kidney and ureter. Surgery. (2019) 37(7):359–64. 10.1016/j.mpsur.2019.04.005

[43] McBride JM. Embryology, Anatomy, and Histology of the Kidney. New York: Springer (2016). p. 1–18.

[44] Moinuddin Z Dhanda R. Anatomy of the kidney and ureter. Anaesth Intensive Care Med. (2015) 16(6):247–52. 10.1016/j.mpaic.2015.04.001

[45] Lafranca JA van Bruggen M Kimenai HJ Tran TC Terkivatan T Betjes MG. Vascular multiplicity should not be a contra-indication for live kidney donation and transplantation. PLoS One. (2016) 11(4):e0153460. 10.1371/journal.pone.0153460 27077904 PMC4831799

[46] Cinar C Turkvatan A. Prevalence of renal vascular variations: evaluation with MDCT angiography. Diagn Interv Imaging. (2016) 97(9):891–7. 10.1016/j.diii.2016.04.001 27178758

[47] Emiroglu R Koseoglu F Karakayali H Bilgin N Haberal M. Multiple-artery anastomosis in kidney transplantation. Transplant Proc. (2000) 32(3):617–9. 10.1016/S0041-1345(00)00919-2 10812141

[48] Osman Y Shokeir A Ali-el-Dein B Tantawy M Wafa EW el-Dein AB. Vascular complications after live donor renal transplantation: study of risk factors and effects on graft and patient survival. J Urol. (2003) 169(3):859–62. 10.1097/01.ju.0000050225.74647.5a 12576799

[49] Humar A Matas AJ. Surgical complications after kidney transplantation. Semin Dial. (2005) 18(6):505–10. 10.1111/j.1525-139X.2005.00097.x 16398714

[50] Haberal M Boyvat F Akdur A Kirnap M Ozcelik U Yarbug Karakayali F. Surgical complications after kidney transplantation. Exp Clin Transplant. (2016) 14(6):587–95. 27934557

[51] Sabharwal S Young B Sabharwal S Brandon K Assem S. A review of literature of a functional, congenital intrathoracic kidney. J Cardiothorac Surg. (2025) 20(1):20. 10.1186/s13019-024-03306-5 39757195 PMC11700456

[52] Ghandour A Partovi S Karuppasamy K Rajiah P. Congenital anomalies of the IVC-embryological perspective and clinical relevance. Cardiovasc Diagn Ther. (2016) 6(6):482–92. 10.21037/cdt.2016.11.18 28123970 PMC5220208

[53] Aristotle S Sundarapandian, Felicia C. Anatomical study of variations in the blood supply of kidneys. J Clin Diagn Res. (2013) 7(8):1555–7. 10.7860/JCDR/2013/6230.3203 24086837 PMC3782894

[54] Valenzuela Fuenzalida JJ Vera-Tapia K Urzúa-Márquez C Yáñez-Castillo J Trujillo-Riveros M Koscina Z. Anatomical variants of the renal veins and their relationship with morphofunctional alterations of the kidney: a systematic review and meta-analysis of prevalence. J Clin Med. (2024) 13(13):3689. 10.3390/jcm13133689 38999255 PMC11242292

[55] Bikauskaitė S Počepavičiūtė K Velička L Jankauskas A Trumbeckas D Šuopytė E. Reconstruction of a lower polar artery for kidney transplantation using donor ovarian vein: case report with literature review. Medicina. (2021) 57(11). 10.3390/medicina57111248 PMC8618098 34833466

[56] Vincenzi P Gonzalez J Guerra G Gaynor JJ Alvarez A Ciancio G. Complex surgical reconstruction of upper pole artery in living-donor kidney transplantation. Ann Transplant. (2021) 26:e926850. 10.12659/AOT.926850 33446626 PMC7814512

[57] Cicek SK Ergun S Akinci O Sariyar M. Renal vascular and ureteral anatomic variations in 1859 potential living renal donors. Transplant Proc. (2021) 53(7):2153–6. 10.1016/j.transproceed.2021.07.030 34404539

[58] Majos M Stefańczyk L Szemraj-Rogucka Z Elgalal M De Caro R Macchi V. Does the type of renal artery anatomic variant determine the diameter of the main vessel supplying a kidney? A study based on CT data with a particular focus on the presence of multiple renal arteries. Surg Radiol Anat. (2018) 40(4):381–8. 10.1007/s00276-017-1930-z 28980056 PMC5880851

[59] Nayak SB Shetty SD Ravindra S Sirasanagandla SR Aithal AP Patil J. Eight prehilar branches of the right renal artery. Anat Cell Biol. (2014) 47(3):214–6. 10.5115/acb.2014.47.3.214 25276483 PMC4178199

[60] Kumaresan M Saikarthik J Sangeetha A Saraswathi I Senthil Kumar K Roselin P. Peri-hilar branching pattern and variations of the renal artery among Indian kidney donors using pre-operative computed tomography angiography: an anatomical study and review. Folia Morphol. (2022) 81(4):971–82. 10.5603/FM.a2021.0103 34642929

[61] Shoja MM Tubbs RS Shakeri A Loukas M Ardalan MR Khosroshahi HT. Peri-hilar branching patterns and morphologies of the renal artery: a review and anatomical study. Surg Radiol Anat. (2008) 30(5):375–82. 10.1007/s00276-008-0342-5 18368282

[62] Graves FT. The anatomy of the intrarenal arteries and its application to segmental resection of the kidney. Br J Surg. (1954) 42(172):132–9. 10.1002/bjs.18004217204 13209036

[63] Fine H Keen EN. The arteries of the human kidney. J Anat. (1966) 100(Pt 4):881–94. 5969982 PMC1270833

[64] Kang WY Sung DJ Park BJ Kim MJ Han NY Cho SB. Perihilar branching patterns of renal artery and extrarenal length of arterial branches and tumour-feeding arteries on multidetector CT angiography. Br J Radiol. (2013) 86(1023):20120387. 10.1259/bjr.20120387 23418206 PMC3608057

[65] Dăescu E Zăhoi DE Motoc A Alexa A Baderca F Enache A. Morphological variability of the renal artery branching pattern: a brief review and an anatomical study. Rom J Morphol Embryol. (2012) 53(2):287–91. 22732797

[66] Mishra GP Bhatnagar S Singh B. Anatomical variations of upper segmental renal artery and clinical significance. J Clin Diagn Res. (2015) 9(8):AC01–3. 10.7860/JCDR/2015/12326.6280 PMC4576523 26435932

[67] Coen LD Raftery AT. Anatomical variations of the renal arteries and renal transplantation. Clin Anat. (1992) 5(6):425–32. 10.1002/ca.980050602

[68] Mishall PL. Renal arteries. In: Tubbs RS Shoja MM Loukas M, editors. Bergman’s Comprehensive Encyclopedia of Human Anatomic Variation. Hoboken: Wiley-Blackwell (2016). p. 682–93.

[69] Cases C Garcia-Zoghby L Manzorro P Valderrama-Canales FJ Munoz M Vidal M. Anatomical variations of the renal arteries: cadaveric and radiologic study, review of the literature, and proposal of a new classification of clinical interest. Ann Anat. (2017) 211:61–8. 10.1016/j.aanat.2017.01.012 28163208

[70] Satyapal KS Haffejee AA Singh B Ramsaroop L Robbs JV Kalideen JM. Additional renal arteries: incidence and morphometry. Surg Radiol Anat. (2001) 23(1):33–8. 10.1007/s00276-001-0033-y 11370140

[71] Yi SQ Ueno Y Naito M Ozaki N Itoh M. The three most common variations of the left renal vein: a review and meta-analysis. Surg Radiol Anat. (2012) 34(9):799–804. 10.1007/s00276-012-0968-1 22535303

[72] Alexopoulos S Matsuoka L Karp SJ. Chapter 37: Surgical management of the renal transplant recipient. In: Himmelfarb J Ikizler TA, editors. Chronic Kidney Disease, Dialysis, and Transplantation, 4th ed. Philadelphia: Elsevier (2019). p. 582–90.e3.

[73] Kok NF Dols LF Hunink MG Alwayn IP Tran KT Weimar W. Complex vascular anatomy in live kidney donation: imaging and consequences for clinical outcome. Transplantation. (2008) 85(12):1760–5. 10.1097/TP.0b013e318172802d 18580468

[74] Hostiuc S Rusu MC Negoi I Dorobanțu B Grigoriu M. Anatomical variants of renal veins: a meta-analysis of prevalence. Sci Rep. (2019) 9(1). 10.1038/s41598-019-47280-8 31346244 PMC6658480

[75] Sonawane GB Moorthy KH Pillai BS. Newer variants of retroaortic left renal vein. Indian J Urol. (2020) 36(2):142–3. 10.4103/iju.IJU_380_19 32549670 PMC7279090

[76] Nam JK Park SW Lee SD Chung MK. The clinical significance of a retroaortic left renal vein. Korean J Urol. (2010) 51(4):276–80. 10.4111/kju.2010.51.4.276 20428432 PMC2858856

[77] Bass JE Redwine MD Kramer LA Huynh PT Harris JH Jr. Spectrum of congenital anomalies of the inferior vena cava: cross-sectional imaging findings. Radiographics. (2000) 20(3):639–52. 10.1148/radiographics.20.3.g00ma09639 10835118

[78] Anjamrooz SH Azari H Abedinzadeh M. Abnormal patterns of the renal veins. Anat Cell Biol. (2012) 45(1):57–61. 10.5115/acb.2012.45.1.57 22536553 PMC3328742

[79] Davis CJ Jr Lundberg GD. Retroaortic left renal vein: a relatively frequent anomaly. Am J Clin Pathol. (1968) 50(6):700–3. 10.1093/ajcp/50.6.700 4881947

[80] Karaman B Koplay M Ozturk E Basekim CC Ogul H Mutlu H. Retroaortic left renal vein: multidetector computed tomography angiography findings and its clinical importance. Acta Radiol. (2007) 48(3):355–60. 10.1080/02841850701244755 17453511

[81] Zhu J Zhang L Yang Z Zhou H Tang G. Classification of the renal vein variations: a study with multidetector computed tomography. Surg Radiol Anat. (2015) 37(6):667–75. 10.1007/s00276-014-1403-6 25567101

[82] Eldefrawy A Arianayagam M Kanagarajah P Acosta K Manoharan M. Anomalies of the inferior vena cava and renal veins and implications for renal surgery. Cent European J Urol. (2011) 64(1):4–8. 10.5173/ceju.2011.01.art1 24578852 PMC3921701

[83] Monkhouse WS Khalique A. The adrenal and renal veins of man and their connections with azygos and lumbar veins. J Anat. (1986) 146:105–15. 3693053 PMC1166527

[84] Janschek EC Rothe AU Holzenbein TJ Langer F Brugger PC Pokorny H. Anatomic basis of right renal vein extension for cadaveric kidney transplantation. Urology. (2004) 63(4):660–4. 10.1016/j.urology.2003.11.010 15072874

[85] Roza AM Perloff LJ Naji A Grossman RA Barker CF. Living-related donors with bilateral multiple renal arteries. A twenty-year experience. Transplantation. (1989) 47(2):397–9. 10.1097/00007890-198902000-00045 2645725

[86] Salehipour M Salahi H Jalaeian H Bahador A Nikeghbalian S Barzideh E. Vascular complications following 1500 consecutive living and cadaveric donor renal transplantations: a single center study. Saudi J Kidney Dis Transpl. (2009) 20(4). 19587495

[87] Zorgdrager M Krikke C Hofker SH Leuvenink HG Pol RA. Multiple renal arteries in kidney transplantation: a systematic review and meta-analysis. Ann Transplant. (2016) 21:469–78. 10.12659/AOT.898748 27470979

[88] Afriansyah A Rasyid N Rodjani A Wahyudi I Mochtar CA Susalit E. Laparoscopic procurement of single versus multiple artery kidney allografts: meta-analysis of comparative studies. Asian J Surg. (2019) 42(1):61–70. 10.1016/j.asjsur.2018.06.001 30042021

[89] Tyson MD Castle EP Ko EY Andrews PE Heilman RL Mekeel KL. Living donor kidney transplantation with multiple renal arteries in the laparoscopic era. Urology. (2011) 77(5):1116–21. 10.1016/j.urology.2010.07.503 21145095

[90] Hsu TH Su L Ratner LE Trock BJ Kavoussi LR. Impact of renal artery multiplicity on outcomes of renal donors and recipients in laparoscopic donor nephrectomy. Urology. (2003) 61(2):323–7. 10.1016/S0090-4295(02)02124-6 12597939

[91] Alomar OSK. Comparison between single and multiple renal vessels in live donor allograft kidney transplantation: surgical aspects and outcomes, 25 years experience. Int J Surg Open. (2021) 35:100394. 10.1016/j.ijso.2021.100394

[92] Benedetti E Troppmann C Gillingham K Sutherland DE Payne WD Dunn DL. Short- and long-term outcomes of kidney transplants with multiple renal arteries. Ann Surg. (1995) 221(4):406–14. 10.1097/00000658-199504000-00012 7726677 PMC1234591

[93] Laouad I Bretagnol A Fabre E Halimi JM Al-Najjar A Boutin JM. Kidney transplant with multiple renal artery grafts from deceased donors: are long-term graft and patient survival compromised? Prog Transplant. (2012) 22(1):102–9. 10.7182/pit2012992 22489451

[94] Troppmann C Wiesmann K McVicar JP Wolfe BM Perez RV. Increased transplantation of kidneys with multiple renal arteries in the laparoscopic live donor nephrectomy era: surgical technique and surgical and nonsurgical donor and recipient outcomes. Arch Surg. (2001) 136(8):897–907. 10.1001/archsurg.136.8.897 11485525

[95] Tiwari B Pandey P Vezhaventhen G Saravanan K. Various techniques and outcomes of arterial anastomosis in live renal transplant: an institutional experience. Cureus. (2022) 14(5):e25262. 10.7759/cureus.25262 35755546 PMC9224832

[96] Leslie SW Sajjad H. Anatomy, abdomen and pelvis, renal artery. In: StatPearls. Treasure Island (FL): StatPearls Publishing LLC (2023). 29083626

[97] Leyba K Wagner B. Gadolinium-based contrast agents: why nephrologists need to be concerned. Curr Opin Nephrol Hypertens. (2019) 28(2):154–62. 10.1097/MNH.0000000000000475 30531473 PMC6416778

[98] Hekimoglu A Ergun O. Evaluation of renal vascular variations with computed tomography. Afr J Urol. (2022) 28(1):21. 10.1186/s12301-022-00290-x

[99] Zhang J Hu X Wang W Li X Hang Y Zhang X. Role of multidetector-row computed tomography in evaluation of living renal donors. Transplant Proc. (2010) 42(9):3433–6. 10.1016/j.transproceed.2010.06.002 21094791

[100] Helmy D Troppmann C Fananapazir G. Preoperative imaging evaluation of living kidney transplant donors. In: Fananapazir G Lamba R, editors. Transplantation Imaging. Cham: Springer International Publishing (2018). p. 17–32.

[101] Kawamoto S Fishman EK. MDCT angiography of living laparoscopic renal donors. Abdom Imaging. (2006) 31(3):361–73. 10.1007/s00261-005-0371-z 16447094

[102] Ghonge NP Gadanayak S Rajakumari V. MDCT evaluation of potential living renal donor, prior to laparoscopic donor nephrectomy: what the transplant surgeon wants to know? Indian J Radiol Imaging. (2014) 24(4):367–78. 10.4103/0971-3026.143899 25489130 PMC4247506

[103] Lype S David S Hilliard S Shaw A Jamieson NV Praseedom RK. When one becomes more: minimum renal artery length in laparoscopic live donor nephrectomy. Clin Transplant. (2015) 29(7):588–93. 10.1111/ctr.12560 25965009

[104] Namasivayam S Kalra MK Small WC Torres WE Mittal PK. Multidetector row computed tomography evaluation of potential living laparoscopic renal donors: the story so far. Curr Probl Diagn Radiol. (2006) 35(3):102–14. 10.1067/j.cpradiol.2006.02.005 16701121

[105] Watson CJE Friend PJ Marson LP. Chapter 11: Surgical techniques of kidney transplantation. In: Knechtle SJ Marson LP Morris PJ, editors. Kidney Transplantation - Principles and Practice, 8th ed. Philadelphia: Elsevier (2019). p. 157–72.

[106] Raman SS Pojchamarnwiputh S Muangsomboon K Schulam PG Gritsch HA Lu DSK. Surgically relevant normal and variant renal parenchymal and vascular anatomy in preoperative 16-MDCT evaluation of potential laparoscopic renal donors. Am J Roentgenol. (2007) 188(1):105–14. 10.2214/AJR.05.1002 17179352

[107] Pandya VK Patel AS Sutariya HC Gandhi SP. Evaluation of renal vascular anatomy in live renal donors: role of multi detector computed tomography. Urol Ann. (2016) 8(3):270–6. 10.4103/0974-7796.184898 27453646 PMC4944617

[108] Hiller J Sroka M Holochek MJ Morrison A Kavoussi LR Ratner LE. Functional advantages of laparoscopic live-donor nephrectomy compared with conventional open-donor nephrectomy. J Transpl Coord. (1997) 7(3):134–40. 10.7182/prtr.1.7.3.v503420j4hr31621 9505658

[109] Davari HR Malek-Hossini SA Salahi H Bahador A Rais-Jalali GA Behzadi S. Sequential anastomosis of accessory renal artery to external iliac artery in the management of renal transplantation with multiple arteries. Transplant Proc. (2003) 35(1):329–31. 10.1016/S0041-1345(02)03838-1 12591426

[110] Doan HQ Nguyen TM Nguyen NQ Pham LH Ninh KV. Angioplasty and angiorrhaphy efficiency in renal transplantation scenarios with multiple arteries and veins. Asian J Surg. (2022) 45(11):2185–90. 10.1016/j.asjsur.2021.11.006 34810116

[111] Barlow AD Nicholson ML. New surgical techniques in transplantation. In: Hakim NS Papalois VE, editors. Abdominal Organ Transplantation. Chichester: Wiley-Blackwell (2013). p. 17–32. 10.1002/9781118483664.ch2

[112] Johnson RJ Floege J Tonelli M. Comphrensive Clinical Nephrology, 7th ed. Philadelphia: Elsevier (2023). p. 10.

[113] Shoskes DA. Kidney transplant recipient surgery. In: Srinivas TR Shoskes DA, editors. Kidney and Pancreas Transplantation: A Practical Guide. Totowa, NJ: Humana Press (2011). p. 211–7.

[114] Iwami D Hotta K Sasaki H Hirose T Higuchi H Takada Y. A 2-mm cutoff value is reasonable and feasible for vascular reconstruction in a kidney allograft with multiple arteries. Transplant Proc. (2019) 51(5):1317–20. 10.1016/j.transproceed.2019.01.137 31027827

[115] Del Gaudio M Amaduzzi A Neri F Ravaioli M. Renal transplantation: surgical technique. In: Pinna AD Ercolani G, editors. Abdominal Solid Organ Transplantation: Immunology, Indications, Techniques, and Early Complications. Cham: Springer International Publishing (2015). p. 283–92.

[116] Panwar P Bansal D Maheshwari R Chaturvedi S Desai P Kumar A. Management of donor kidneys with double renal arteries with significant luminal discrepancy: a retrospective cohort study. Indian J Urol. (2020) 36(3):200–4. 10.4103/iju.IJU_196_20 33082635 PMC7531379

[117] Humar A Sturdevant ML. Atlas of Organ Transplantation. London, United Kingdom: Springer London, Limited (2015).

[118] Kadotani Y Okamoto M Akioka K Ushigome H Ogino S Nobori S. Renovascular reconstruction of grafts with renal artery variations in living kidney transplantation. Transplant Proc. (2005) 37(2):1049–51. 10.1016/j.transproceed.2005.01.033 15848619

[119] Singh PB Goyal NK Kumar A Dwivedi US Trivedi S Singh DK. Renal transplantation using live donors with vascular anomalies: a salvageable surgical challenge. Saudi J Kidney Dis Transpl. (2008) 19(4):554–8. 18580012

[120] Sakpal SV Hardie K Peters E Saucedo-Crespo H. “Triple V-plasty”: creation of a single orifice for three renal arteries in live-donor transplantation. Transplant Proc. (2023) 55:1900–2. 10.1016/j.transproceed.2023.03.094 37479542

[121] Aulakh BS Singh SK Kaur N Singh A Grewal A Kumar M. Three-legged pair of pants anastomosis: a rare technique for renal transplantation. Transplant Proc. (2004) 36(7):2191–3. 10.1016/j.transproceed.2004.09.002 15518797

[122] Pal DK Sanki PK Roy S. Analysis of outcome of end-to-end and end-to-side internal iliac artery anastomosis in renal transplantation: our initial experience with a case series. Urol Ann. (2017) 9(2):166–9. 10.4103/0974-7796.204176 28479769 PMC5405661

[123] Mohamed IH Bagul A Doughman T Nicholson ML. Use of internal iliac artery as a side-to-end anastomosis in renal transplantation. Ann R Coll Surg Engl. (2012) 94(1):e36–7. 10.1308/003588412X13171221499540 22524924 PMC3954224

[124] Abdel-Hamid IA Eraky I Fouda MA Mansour OE. Role of penile vascular insufficiency in erectile dysfunction in renal transplant recipients. Int J Impot Res. (2002) 14(1):32–7. 10.1038/sj.ijir.3900810 11896475

[125] Matheus WE Reis LO Ferreira U Mazzali M Denardi F Leitao VA. Kidney transplant anastomosis: internal or external iliac artery? Urol J. (2009) 6(4):260–6. 20027554

[126] Danovitch GM. Handbook of Kidney Transplantation. Philadelphia, United States: Wolters Kluwer (2017).

[127] Gerstenkorn C Papalois VE Thomusch O Maxwell AP Hakim N. Surgical management of multiple donor veins in renal transplantation. Int Surg. (2006) 91(6):345–7. 17256434

[128] Hoff M Leighton P Hosgood SA Nicholson ML. Anastomosis of dual renal transplant veins. J Surg Case Rep. (2020) 2020(9):rjaa310. 10.1093/jscr/rjaa310 32963761 PMC7490215

[129] Mang J Hennig L Biernath N Liefeldt L Bichmann A Ralla B. Is a retroaortic vein a risk factor in laparoscopic living donor nephrectomy? Urol Int. (2020) 104(7–8):641–5. 10.1159/000507642 32417839 PMC7592925

[130] Patil AB Javali TD Nagaraj HK Babu S Nayak A. Laparoscopic donor nephrectomy in unusual venous anatomy - donor and recepient implications. Int Braz J Urol. (2017) 43(4):671–8. 10.1590/s1677-5538.ibju.2016.0309 28379667 PMC5557443

